# How adaptation shapes spike rate oscillations in recurrent neuronal networks

**DOI:** 10.3389/fncom.2013.00009

**Published:** 2013-02-27

**Authors:** Moritz Augustin, Josef Ladenbauer, Klaus Obermayer

**Affiliations:** ^1^Department of Software Engineering and Theoretical Computer Science, Technische Universität BerlinBerlin, Germany; ^2^Bernstein Center for Computational Neuroscience BerlinBerlin, Germany

**Keywords:** spike frequency adaptation, adaptation, oscillations, rate models, network dynamics, Fokker–Planck, mean-field, recurrent network

## Abstract

Neural mass signals from *in-vivo* recordings often show oscillations with frequencies ranging from <1 to 100 Hz. Fast rhythmic activity in the beta and gamma range can be generated by network-based mechanisms such as recurrent synaptic excitation-inhibition loops. Slower oscillations might instead depend on neuronal adaptation currents whose timescales range from tens of milliseconds to seconds. Here we investigate how the dynamics of such adaptation currents contribute to spike rate oscillations and resonance properties in recurrent networks of excitatory and inhibitory neurons. Based on a network of sparsely coupled spiking model neurons with two types of adaptation current and conductance-based synapses with heterogeneous strengths and delays we use a mean-field approach to analyze oscillatory network activity. For constant external input, we find that spike-triggered adaptation currents provide a mechanism to generate slow oscillations over a wide range of adaptation timescales as long as recurrent synaptic excitation is sufficiently strong. Faster rhythms occur when recurrent inhibition is slower than excitation and oscillation frequency increases with the strength of inhibition. Adaptation facilitates such network-based oscillations for fast synaptic inhibition and leads to decreased frequencies. For oscillatory external input, adaptation currents amplify a narrow band of frequencies and cause phase advances for low frequencies in addition to phase delays at higher frequencies. Our results therefore identify the different key roles of neuronal adaptation dynamics for rhythmogenesis and selective signal propagation in recurrent networks.

## Introduction

A prominent characteristic of cortical activity is its rhythmicity as shown by electroencephalography or the local field potential. Dominant oscillation frequencies in these signals range from <1 to 100 Hz and reflect synchronous activity of populations of neurons. Such oscillations are linked to behavioral states (Wang, 2010) and involved in a variety of cognitive functions (Engel et al., 2001; Fries, 2001; Melloni et al., 2007; Ghazanfar et al., 2008; Wang, 2010) as well as pathological conditions (Hammond et al., 2007; Zijlmans et al., 2009; Uhlhaas and Singer, 2010). It is therefore important to understand the mechanisms of oscillations in neuronal networks, how they are initiated and terminated, and how their frequency is determined.

Fast rhythmic activity in the beta and gamma band (>20 Hz) can be generated by network-based mechanisms, such as synaptic excitation-inhibition loops or by feedback inhibition alone (Isaacson and Scanziani, 2011). In these scenarios the oscillation frequency is largely determined by the inhibitory decay time constant (Brunel and Wang, 2003; Tiesinga and Sejnowski, 2009). Low-frequency oscillations, on the other hand, could depend on slow transmembrane outward currents (Compte et al., 2003; Gigante et al., 2007b; Destexhe, 2009), which are mediated by low-threshold voltage-dependent muscarinic (M) and high-threshold calcium-gated afterhyperpolarization (AHP) K^+^ channels, respectively (Brown and Adams, 1980; Connors et al., 1982; Stocker, 2004). These currents cause spike frequency adaptation and are typically more pronounced in cortical regular spiking pyramidal (excitatory) neurons compared to fast spiking (inhibitory) interneurons (La Camera et al., 2006). Both, the M and AHP type K^+^ currents, are susceptible to cholinergic modulation (McCormick, 1992). Their kinetic time constants range from milliseconds to seconds (Abel et al., 2004; Manuel et al., 2005) and can be pharmacologically manipulated (Pedarzani et al., 2001).

Here we study the interplay of the dynamics of such adaptation currents with synaptic excitation and inhibition in recurrent networks of excitatory and inhibitory neurons. Specifically, we ask (1) how adaptation can generate slow oscillations, (2) how it modulates faster rhythms based on synaptic interaction, and (3) how adaptation affects resonance properties of the network.

*In-vivo* recordings from behaving animals have revealed that even when the population activity oscillates, the spike trains of the constituent neurons are rather irregular and display Poisson-like characteristics (Fries, 2001; Wang, 2010). This stochasticity in neuronal responses allows us to derive a mean-field model from a recurrent network of adaptive spiking model neurons coupled through conductance-based synapses with heterogeneous strengths and delays. Our approach is based on the Fokker–Planck (FP) formalism (Brunel, 2000; Deco et al., 2008) and efficiently describes the activity of large networks where the features of the spiking neurons (i.e., the model parameters) are retained. Using this method we analyze network responses to constant as well as rhythmic external input. In particular we describe asynchronous irregular states with constant steady-state activity as well as oscillatory states and their properties. We validate our mean-field results qualitatively by large-scale network simulations.

## Methods

We first describe our network model containing two populations (excitatory and inhibitory) of adaptive spiking neurons with delayed conductance-based synaptic coupling. Based on that model we then derive mean-field model equations and solve them numerically to obtain distributions of the membrane potentials and instantaneous spike rates.

### Network model

We consider a network of *N* = *N*_ℰ_ + *N*_ℐ_ adaptive exponential integrate-and-fire neurons (aEIF) proposed by Brette and Gerstner (2005), where *N*_ℰ_ and *N*_ℐ_ are the numbers of excitatory and inhibitory neurons, respectively. The dynamics of the *i*-th neuron of population α ∈ {ℰ, ℐ} is described by

(1) $$C\frac{dV_{i}^{\alpha }}{dt}=I_{\text{ion}}(V_{i}^{\alpha })-w_{i}^{\alpha }+I_{\text{syn},\;i}^{\alpha }(V_{i}^{\alpha },\;t)$$

(2) $$\tau_{w}\frac{dw_{i}^{\alpha }}{dt}=a(V_{i}^{\alpha }-E_{L})-w_{i}^{\alpha }$$

with reset condition

(3) $$\text{if }V_{i}^{\alpha }>V_{\text{cut}}\text{ then }\left\{ \begin{array}{l} V_{i}^{\alpha }:=V_{r}\\ w_{i}^{\alpha }:=w_{i}^{\alpha }+b. \end{array}\right.$$

The first Equation (1) is for the membrane potential *V*^α^_*i*_, where the capacitive current through the membrane with capacitance *C* equals the sum of ionic currents *I*_ion_, the adaptation current *w*^α^_*i*_ and the synaptic current *I*^α^_syn, *i*_. The ionic currents are given by

(4) $$I_{\text{ion}}(V):=g_{L}(E_{L}-V)+g_{L}\Delta_{T}e^{\frac{V-V_{T}}{\Delta_{T}}},$$

where the first term on the right-hand side describes an Ohmic leak current with conductance *g*_*L*_ and reversal potential *E*_*L*_. The exponential term with threshold slope factor Δ_*T*_ and threshold potential *V*_*T*_ approximates the Na^+^-current which is responsible for the generation of spikes, assuming that the activation of Na^+^-channels is instantaneous and neglecting their inactivation (Fourcaud-Trocme et al., 2003). Equation (2) governs the dynamics of the adaptation current *w*^α^_*i*_, where τ_*w*_ denotes the adaptation time constant and *a* quantifies a conductance that mediates subthreshold adaptation. A spike is said to occur at the time when *V*^α^_*i*_ diverges to infinity, but in practice a finite “cutoff” value *V*_cut_ is chosen. When *V*^α^_*i*_ crosses *V*_cut_ from below, *V*^α^_*i*_ is set to the reset potential *V*_*r*_ and *w*^α^_*i*_ is incremented by *b*, cf. condition (3). In this way spike-triggered adaptation is included in the model. Immediately after the reset, *V*^α^_*i*_ and *w*^α^_*i*_ are clamped for a refractory period *T*_ref_.

The aEIF model has been shown to reproduce a broad range of subthreshold dynamics (Touboul and Brette, 2008) and spike patterns of cortical neurons (Naud et al., 2008) and can well predict their spike times (Jolivet et al., 2008) and post-stimulus time histograms (Pospischil et al., 2011). Importantly, the subthreshold and spike-triggered adaptation components of this model have been shown to capture the effects of the M and AHP currents in a detailed biophysical neuron model, respectively (Ladenbauer et al., 2012).

**Figure 1:**
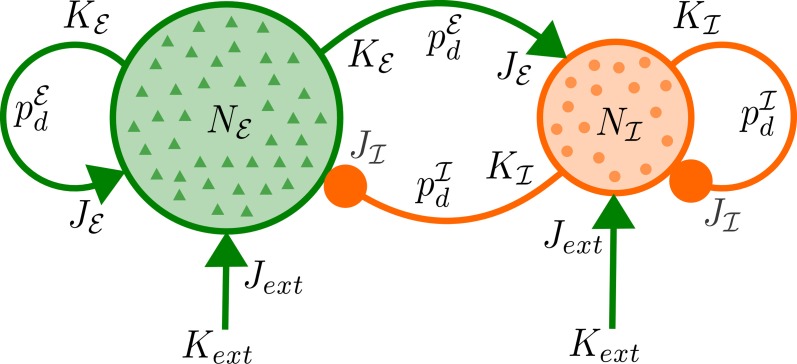
**Network architecture.** Each of *N*_ℰ_ excitatory and *N*_ℐ_ inhibitory neurons receives excitatory input from *K*_ext_ external neurons with mean synaptic strength *J*_ext_ as well as synaptic input from *K*_ℰ_ (*K*_ℐ_) excitatory (inhibitory) neurons of the network with mean strength *J*_ℰ_ (*J*_ℐ_) and delays distributed according to *p*^ℰ^_*d*_ (*p*^ℐ^_*d*_).

Neuron *i* of population α receives total synaptic current

(5) $$I_{\text{syn},\;i}^{\alpha }(V_{i}^{\alpha },\;t):=\sum_{j}I_{ij}^{\alpha ,\text{ ext}}+\sum_{j}I_{ij}^{\alpha ,\;ℰ}+\sum_{j}I_{ij}^{\alpha ,\;ℐ},$$

which is the superposition of synaptic inputs *I*^α, ext^_*ij*_ from *K*_ext_ external excitatory neurons, *I*^α, ℰ^_*ij*_ from *K*_ℰ_ excitatory neurons of the network and *I*^α, ℰ^_*ij*_ from *K*_ℐ_ inhibitory neurons of the network. *j* is the index of the respective presynaptic neuron. The synaptic current *I*^α, γ^_*ij*_ caused by neuron *j* of population γ ∈ {ext, ℰ, ℐ} is modeled using delta functions,

(6) $$I_{ij}^{\alpha ,\text{ ext}}(V_{i}^{\alpha },\;t):=CJ_{ij}^{\alpha ,\text{ ext}}\sum_{k}\delta (t-t_{j}^{k})\left(E_{ℰ}-V_{i}^{\alpha }\right)$$

(7) $$I_{ij}^{\alpha ,\;\beta }(V_{i}^{\alpha },\;t):=CJ_{ij}^{\alpha ,\;\beta }\sum_{k}\delta (t-t_{j}^{k}-d_{ij}^{\alpha ,\beta })\left(E_{\beta }-V_{i}^{\alpha }\right),$$

where β ∈ {ℰ, ℐ} denotes the presynaptic population. *J*^α, γ^_*ij*_ are dimensionless synaptic efficacies drawn from a Gaussian distribution with mean *J*_α, γ_ and standard deviation Δ*J*_α, γ_. Here we consider that *J*_α, γ_ ≡ *J*_γ_ and Δ*J*_α, γ_ ≡ Δ*J*_γ_ depend only on the presynaptic population γ. *t*^*k*^_*j*_ is the *k*-th spike time of neuron *j* from the respective population. *E*_ℰ_ and *E*_ℐ_ denote the excitatory and inhibitory reversal potentials, respectively. *d*^α, β^_*ij*_ is the synaptic delay, sampled using a bi-exponential probability density

(8) $$p_{d}^{\alpha ,\;\beta }(d):=\frac{1}{\tau_{d}-\tau_{r}}\left(e^{-\frac{d-d_{0}}{\tau_{d}}}-e^{-\frac{d-d_{0}}{\tau_{r}}}\right)$$

for positive delays *d*, where *d*_0_ is the minimal delay and τ_*r*_, τ_*d*_ are the rise and decay time constants, for each pair of populations. In the model we use two different delay distributions *p*^ℰ^_*d*_ and *p*^ℐ^_*d*_ which do not depend on the postsynaptic population as for the synaptic weights. For a schematic diagram of the network, see Figure 1.

We assume the neurons from the external population generate spike times according to Poisson processes with rates *r*^α^_ext_(*t*). The spike rate of each population α ∈ {ℰ, ℐ} at time *t* is given by the average number of spikes of neurons from the corresponding population in the interval [*t*, *t* + Δ*t*],

(9) $$r_{\alpha }^{\Delta t}(t):=\frac{1}{N_{\alpha }\Delta t}\sum_{j=1}^{N_{\alpha }}\int_{t}^{t+\Delta t}\sum_{k}\delta (s-t_{j}^{k})ds.$$

In the mean-field limit *N* → ∞, Δ*t* → 0 we obtain a continuous population spike rate *r*_α_(*t*) (see below).

We selected the following parameters for the neuron model: *C* = 200 pF, *g*_*L*_ = 10 nS, *E*_*L*_ = −70 mV, Δ_*T*_ = 1 mV, *V*_*T*_ = −50 mV, *V*_*r*_ = −70 mV, *V*_cut_ = −40 mV, and *T*_ref_ = 1.4 ms (Badel et al., 2008; Destexhe, 2009). For excitatory neurons the adaptation parameters were varied within reasonable ranges: τ_*w*_ ∈ [5, 1000] ms, *a* ∈ [0, 10] nS, *b* ∈ [0, 50] pA. For inhibitory neurons adaptation was neglected (*a* = b = 0) since it was found to be weak in fast spiking interneurons compared to pyramidal cells (La Camera et al., 2006).

The network parameter values were *N*_ℰ_ = 40,000, *N*_ℐ_ = 10,000, *K*_ext_ = 1600, *K*_ℰ_ = 1600, *K*_ℐ_ = 400, *E*_ℰ_ = 0 mV, *E*_ℐ_ = −80 mV, *J*_ext_ = 0.003, *J*_ℰ_ = 0.003, and Δ*J*_γ_ = 0.1*J*_γ_ with γ ∈ {ext, ℰ, ℐ} (Brunel and Wang, 2003). To adjust the balance of recurrent synaptic excitation and inhibition we introduce the parameter

(10) $$g:=\frac{J_{ℐ}|E_{L}-E_{ℐ}|}{J_{ℰ}|E_{L}-E_{ℰ}|},$$

which is the ratio of total charges induced at rest (Kumar et al., 2008). *g* determines *J*_ℐ_ and thus Δ*J*_ℐ_ for fixed *J*_ℰ_ and was varied in [0.8, 4] which yields a physiological range of inhibitory postsynaptic potential amplitudes (Tamas et al., 1997). Note that the value of *g* that corresponds to balanced mean recurrent excitatory and inhibitory synaptic currents depends on the mean membrane potential for each population. The effect of a spike of presynaptic neuron *j* on neuron *i* is mediated by a delayed instantaneous increment or decrement of the postsynaptic membrane potential, cf. Equations (1), (5), and (7). This implies that *d*^α, β^_*ij*_ reflects the conduction delay as well as delays in the synaptic kinetics. We therefore chose the parameter values of *p*^ℰ^_*d*_ and *p*^ℐ^_*d*_ such that conduction delays as well as typical time courses of excitatory AMPA and inhibitory GABA_A_ synaptic receptors are taken into account. The values we selected were *d*_0_ = 1 ms, τ^ℰ^_*r*_ ∈ [1.25, 1.5] ms, τ^ℰ^_*d*_ ∈ [1.5, 2] ms, τ^ℐ^_*r*_ ∈ [0.55, 1.25] ms, and τ^ℐ^_*d*_ ∈ [1.5, 5] ms. The input rate of the excitatory population *r*^ℰ^_ext_ was varied in [1, 12.5] Hz. *r*^ℐ^_ext_ was chosen such that *r*_ℰ_ = r_ℐ_ in case of uncoupled populations of neurons, i.e., *J*_ℰ_ = *J*_ℐ_ = 0.

### Mean-field model

We reduce the two-population network of aEIF neurons to the mean-field model in three steps. First, we replace the synaptic current fluctuations by a Gaussian white noise process via the diffusion approximation. Next, we take a mean-field limit to formulate the stochastic network model in terms of two coupled deterministic scalar partial differential equations (PDE). Finally, to allow for efficient numerical computation we reduce the number of variables in these equations using an adiabatic approximation.

#### Diffusion approximation

We approximate the total synaptic current *I*^α^_syn, *i*_ of Equation (5) by its mean plus a fluctuating Gaussian part, which is justified by the following physiologically plausible assumptions: (1) The number of synaptic inputs to a neuron is large, i.e., *K*_ext_, *K*_ℰ_, *K*_ℐ_ » 1 (Destexhe et al., 2003) and (2) the postsynaptic potential amplitudes elicited by individual presynaptic spikes are small, i.e., *J*_ext_|*E*_ℰ_ − *V*|, *J*_ℰ_|*E*_ℰ_ − *V*|, *J*_ℐ_|*E*_ℐ_ − *V*| « *V*_cut_ − *V*_*r*_ (Williams and Stuart, 2002). We further assume that (3) the network connectivity is random and sparse, i.e., *K*_ℰ_, *K*_ℐ_ « N, and that (4) presynaptic spike times are represented by Poisson processes which are homogeneous in each small time interval. The total synaptic current can then be written as (Brunel, 2000; Nykamp and Tranchina, 2000; Renart et al., 2004; Richardson, 2004; Gigante et al., 2007b)

(11) $$I_{\text{syn},\;i}^{\alpha }\approx \mu_{\alpha ,\;i}(V_{i}^{\alpha },\;t)+\sigma_{\alpha ,\;i}(V_{i}^{\alpha },\;t)\eta_{i}(t),$$

where μ_α, *i*_ and σ_α, *i*_ are the infinitesimal mean and standard deviation of *I*^α^_syn, *i*_, respectively, and η_*i*_ is a Gaussian white noise process with δ-autocorrelation. The infinitesimal mean is given by

(12) $$\begin{array}{l} \mu_{\alpha ,\;i}:=\lim_{\Delta t\to 0}\frac{\langle \int_{t}^{t+\Delta t}I_{\text{syn},\;i}^{\alpha }(s)ds\rangle }{\Delta t}\\ \;=\mu_{\alpha ,\;i}^{\text{ext}}+\mu_{\alpha ,\;i}^{ℰ}+\mu_{\alpha ,\;i}^{ℐ} \end{array}$$

with

(13) $$\mu_{\alpha ,\;i}^{\text{ext}}=C(E_{ℰ}-V_{i}^{\alpha })J_{\text{ext}}K_{\text{ext}}r_{\text{ext}}^{\alpha }(t)$$

(14) $$\mu_{\alpha ,\;i}^{\beta }=C(E_{\beta }-V_{i}^{\alpha })J_{\beta }K_{\beta }(r_{\beta }*p_{\beta })(t),$$

where 〈·〉 denotes the expectation operator. The infinitesimal variance is

(15) $$\begin{array}{l} \sigma_{\alpha ,\;i}^{2}:=\lim_{\Delta t\to 0}\frac{\langle {\left(\int_{t}^{t+\Delta t}I_{\text{syn},\;i}^{\alpha }(s)ds\right)}^{2}\rangle +O(\Delta t^{2})}{\Delta t}\\ \;={\left(\sigma_{\alpha ,\;i}^{\text{ext}}\right)}^{2}+{\left(\sigma_{\alpha ,\;i}^{ℰ}\right)}^{2}+{\left(\sigma_{\alpha ,\;i}^{ℐ}\right)}^{2} \end{array}$$

with

(16) $$\sigma_{\alpha ,\;i}^{\text{ext}}=C(E_{ℰ}-V_{i}^{\alpha })\sqrt{\left(J_{\text{ext}}^{2}+\Delta J_{\text{ext}}^{2}\right)K_{\text{ext}}r_{\text{ext}}(t)}$$

(17) $$\sigma_{\alpha ,\;i}^{\beta }=C(E_{\beta }-V_{i}^{\alpha })\sqrt{\left(J_{\beta }^{2}+\Delta J_{\beta }^{2}\right)K_{\beta }(r_{\beta }*p_{\beta })(t)},$$

where β ∈ {ℰ, ℐ} and * denotes convolution. In Equations (13), (14), (16), and (17) we have used that the presynaptic Poisson processes, the synaptic weights and delays are independent.

#### Mean-field limit

We analyze networks of sparsely coupled neurons, i.e., the probability for a connection between any pair of neurons is low, cf. assumption (3) above. For large *N* correlations between the fluctuations of synaptic currents of different neurons become negligible, i.e., 〈 η_*i*_(*t*) η_*j*_(*t*) 〉 = 0 for *i* ≠ *j*. In the mean-field limit *N* → ∞ the network model Equations (1)–(4), Equations (11)–(17) can be described by two FP equations—one for each population α—which are delay-coupled by the population spike rates *r*_ℰ_ and *r*_ℐ_,

(18) $$\frac{\partial p_{\alpha }}{\partial t}+\frac{\partial S_{\alpha }^{V}}{\partial V}+\frac{\partial S_{\alpha }^{w}}{\partial w}=0$$

with

(19) $$S_{\alpha }^{V}:=\left(\frac{I_{\text{ion}}(V)-w+\mu_{\alpha }}{C}-\frac{\sigma_{\alpha }}{2C^{2}}\frac{\partial \sigma_{\alpha }}{\partial V}\right)p_{\alpha }-\frac{\sigma_{\alpha }^{2}}{2C^{2}}\frac{\partial p_{\alpha }}{\partial V}$$

(20) $$S_{\alpha }^{w}:=\frac{a(V-E_{L})-w}{\tau_{w}}p_{\alpha }.$$

*p*_α_(*V*, *w*, *t*) is the probability density to find a neuron of population α in the state (*V*, *w*) at time *t*. *S*^*V*^_α_(*V*, *w*, *t*) and *S*^*w*^_α_(*V*, *w*, *t*) are the probability fluxes in positive *V* and *w* direction, respectively. Note that we used the Stratonovich interpretation of the underlying stochastic equations (Risken, 1996; Richardson, 2004). To account for the reset condition (3) the flux through the cutoff voltage *V*_cut_ at *w* is re-injected after the refractory period *T*_ref_ at *V*_*r*_, *w* + *b*, i.e.,

(21) $$\lim_{V↓V_{r}}S_{\alpha }^{V}(V,\;w+b,\;t)-\lim_{V↑V_{r}}S_{\alpha }^{V}(V,\;w+b,\;t)=S_{\alpha }^{V}(V_{\text{cut}},\;w,\;t-T_{\text{ref}})\;\forall w\in \mathbb{R}.$$

This implies that in general *p*_α_ is not differentiable at the line *V* = *V*_*r*_. The boundary conditions are reflecting for *w* → ±∞, *V* → −∞ and absorbing for *V* = *V*_cut_,

(22) $$\lim_{w\to \pm \infty }S_{\alpha }^{w}(V,\;w)=0\;\forall V\in (-\infty ,\;V_{\text{cut}}]$$

(23) $$\lim_{V\to -\infty }S_{\alpha }^{V}(V,\;w)=0\;\forall w\in \mathbb{R}$$

(24) $$p_{\alpha }(V_{\text{cut}},\;w)=0\;\forall w\in \mathbb{R}$$

The spike rate of population α is given by the integral of the cutoff fluxes,

(25) $$r_{\alpha }(t)=\int_{\mathbb{R}}S_{\alpha }^{V}(V_{\text{cut}},\;w,\;t)dw.$$

At any timepoint *t* the histogram of the membrane potentials of neurons in population α can be seen as a sample drawn from the probability density *p*_α_(*V*, *t*) which is governed by the FP equation.

#### Adiabatic approximation

Solving the 2 + 1 dimensional PDE (Equations 18–20) with corresponding reset and boundary conditions (21)–(24) numerically is possible but computationally demanding. We therefore reduce the dimensionality of the FP system Equations (18)–(20) assuming the timescales of membrane voltage and adaptation current dynamics are separable. This is justified by the observation that the dynamics of neuronal adaptation is significantly slower than the other in the model system such as membrane time constant and average inter-spike interval (Womble and Moises, 1992; Stocker, 2004). Under this assumption, the adaptation current of each neuron can be seen as an efficient integrator that filters the fluctuations in the neuronal activity. We approximate *w*^α^_*i*_(*t*) in Equation (2) by its population average *w*_α_(*t*), which evolves according to

(26) $$\tau_{w}\frac{dw_{\alpha }}{dt}=a({\langle V\rangle }_{p_{\alpha }(V,\;t)}-E_{L})-w_{\alpha }+\tau_{w}br_{\alpha }(t),$$

where 〈·〉_*p*_ denotes the average over the density *p* (Brunel et al., 2003; Gigante et al., 2007b). The probability density *p*_α_(*V*, *t*) then satisfies the 1 + 1 dimensional FP equation

(27) $$\frac{\partial p_{\alpha }}{\partial t}+\frac{\partial S_{\alpha }^{V}}{\partial V}=0,$$

where again *S*^*V*^_α_ is the probability flux defined in Equation (19) and *w* : = *w*_α_(*t*) appears as a system parameter. The reset condition is

(28) $$\lim_{V↓V_{r}}S_{\alpha }^{V}(V,\;t)-\lim_{V↑V_{r}}S_{\alpha }^{V}(V,\;t)=S_{\alpha }^{V}(V_{\text{cut}},\;t-T_{\text{ref}}).$$

and the boundary conditions (23)–(24) become

(29) $$\lim_{V\to -\infty }S_{\alpha }^{V}(V)=0,$$

(30) $$p_{\alpha }(V_{\text{cut}})=0.$$

The population spike rates are given by the corresponding fluxes through the cutoff voltage,

(31) $$r_{\alpha }(t)=S_{\alpha }^{V}(V_{\text{cut}},\;t).$$

Note that the adiabatic approximation described above could be applied repeatedly for additional slow variables.

### Numerical solution

We solved the reduced FP Equation (27) subject to conditions (28)–(30) and mean adaptation current dynamics (Equation 26) forward in time until either steady states *r*^∞^_ℰ_, *r*^∞^_ℐ_ with *r*^∞^_α_ : = lim_*t* → ∞_ *r*_α_(*t*) or stable oscillatory states were reached. The probability densities *p*_ℰ_, *p*_ℐ_ were initialized using normalized Gaussians with mean 0.5 · (*V*_*r*_ + *V*_*T*_) and standard deviation 0.2 · (*V*_*T*_ − *V*_*r*_). We applied a first-order finite volume method on a finite and non-uniform grid *V*_0_ < *V*_1_ < ··· < *V*_*N*_*V*__ using upwind-fluxes to stabilize the numerical solution (LeVeque, 2002). Time was discretized using the implicit Euler method on an equidistant grid, i.e., $t_{n+1}-t_{n}\equiv \bar{\Delta t}$. The resulting linear equation systems were solved with a preconditioned Krylov subspace method in each time step. Specifically, BiCGSTAB (van der Vorst, 1992) was used in combination with an incomplete LU decomposition preconditioner (Saad, 2003) that strongly improved the convergence speed.

*w*_ℰ_ was initialized with values *w*_ℰ_(0) ∈ [0, 500] pA (and *w*_ℐ_ ≡ 0). The other parameters were $\bar{\Delta t}=50\text{ μs}$, min_*m*_ Δ*V*_*m*_ = 1 μ*V* with Δ*V*_*m*_ : = *V*_*m* + 1_−*V*_m_, *V*_0_ : = −100 μV, *V*_*N*_*V*__ = *V*_cut_ and *N*_*V*_ = 256.

We complemented the mean-field results with numerical simulations of the network model Equations (1)–(4) using a Runge–Kutta second order method implemented in Brian 1.4 (Goodman and Brette, 2009) with a time step of 50 μs.

In case of stable periodic population spike rates the oscillation frequency was determined by the dominant frequency of the Fourier spectrum of *r*_ℰ_ over the last 2 s of runtime.

## Results

### Adaptation mediates oscillations

**Figure 2:**
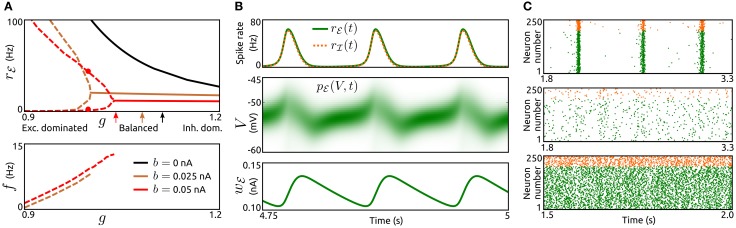
**Population bursts caused by spike-triggered adaptation. (A)** Top: Spike rate *r*_ℰ_ of the excitatory population as a function of the strength of inhibition *g* for networks without spike-triggered adaptation (*b* = 0, black) and with increased levels of *b* (0.025 nA, brown and 0.05 nA, red). In case of stable oscillatory states the maxima and minima of the periodic *r*_ℰ_ are shown by dashed lines. Solid lines represent asynchronous states. Arrows indicate balance of recurrent excitation and inhibition for both populations. Bottom: Corresponding oscillation frequencies *f*. τ_*w*_ = 200 ms, *a* = 0, and *r*^ℰ^_ext_ = 6.25 Hz. The parameter values for both delay distributions *p*^ℰ^_*d*_, *p*^ℐ^_*d*_ were τ_*r*_ = 1.5 ms and τ_*d*_ = 2 ms. For other model parameters see the section Methods. **(B)**, Top: Time-dependent spike rates *r*_ℰ_(*t*) (green) and *r*_ℐ_(*t*) (orange, dashed) for the parameter values *b* = 0.05 nA and *g* = 1, as indicated in **(A)** by red dots. Center: Corresponding membrane potential density *p*_ℰ_(*V*, *t*). Bottom: Corresponding mean adaptation current *w*_ℰ_(*t*). **(C)**: Raster plots of simulated networks of *N* = 50,000 aEIF neurons for *b* = 0.05 nA, *g* = 0.85 (top), *b* = 0.05 nA, *g* = 1.05 (center) and *b* = 0, *g* = 1 (bottom). The spike times of 200 excitatory neurons and 50 inhibitory neurons, all randomly selected, are shown by green and orange dots, respectively. τ_*w*_ = 200 ms, *a* = 0, and *r*^ℰ^_ext_ = 3.75 Hz. Other parameter values as in **(A)**.

To examine how the interplay of adaptation and recurrent synaptic input shapes network dynamics we vary the type, strength and timescale (parameters *a*, *b*, and τ_*w*_) of adaptation for excitatory neurons as well as the strength of synaptic inhibition (parameter *g*) across networks. Adaptation currents are disregarded for inhibitory neurons, which is supported by experimental observations, see the section Methods. We consider constant rates *r*^ℰ^_ext_, *r*^ℐ^_ext_ for the external Poisson-inputs and identical delay distributions *p*^ℰ^_*d*_ ≡ *p*^ℐ^_*d*_. First, we examine steady-state spike rates, oscillation amplitudes and frequencies for networks with different values of spike-triggered adaptation *b* and inhibition strength *g*, see Figure 2A. All networks without adaptation (*a* = *b* = 0) settle into asynchronous states with constant population rates that decrease with increasing *g*. For networks with increased *b* slow oscillatory states become stable if recurrent excitation is sufficiently strong. The larger *b* is, the less recurrent excitation is necessary for sustained oscillations. Amplitude and period of the oscillatory rate decrease with an increase of *b* and *g*, respectively. Thus, in networks where recurrent synaptic excitation dominates inhibition at least slightly, spike-triggered adaptation *b* generates spike rate oscillations. The dynamics of an example network is shown in Figure 2B. The evolution of the population spike rates *r*_ℰ_, *r*_ℐ_, membrane potential probability densities *p*_ℰ_, *p*_ℐ_ and adaptation current *w*_ℰ_ display periodic bursts of population activity. As a validation of the findings above using the mean-field model the activity of simulated large networks of spiking neurons is shown in Figure 2C. The raster plots reveal population bursts when *b* is increased and *g* is small. An asynchronous state with low population activity occurs if *g* is increased. If in addition adaptation is removed (*a* = *b* = 0) the network settles into an asynchronous state with increased spike rates.

The mechanism that generates these oscillations is a loop of recurrent excitation, build up and decay of adaptation current as indicated in Figure 2B. A low level of population activity is initiated by the external input *r*^ℰ^_ext_ and recurrent synaptic excitation boosts the activity, thereby increasing the adaptation current *w*_ℰ_ through *b* in a spike rate dependent way. The adaptation current in turn acts as a negative feedback which eventually outweighs the recurrent excitation. The population activity drops rapidly and the adaptation current decays slowly. Upon recovery from the adaptation current the cycle starts again.

**Figure 3:**
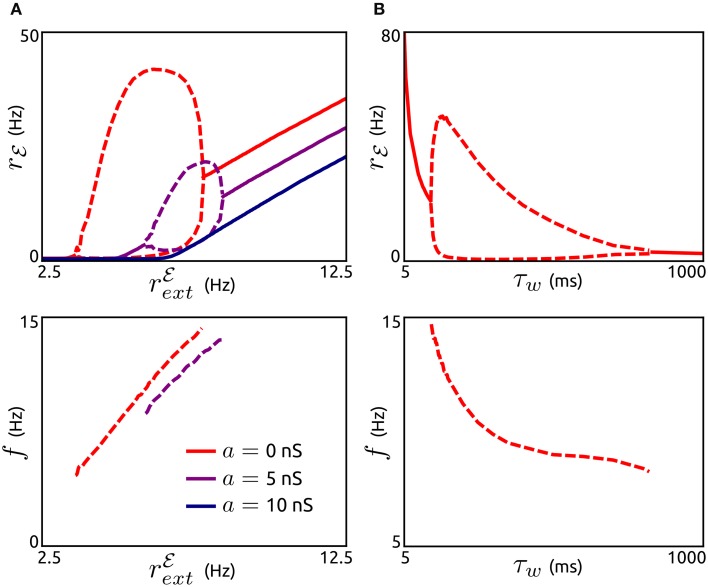
**Effects of subthreshold adaptation, external input, and adaptation timescale on population bursts. (A)**, Top: Spike rate *r*_ℰ_ depending on the external input *r*^ℰ^_ext_ for networks without subthreshold adaptation (*a* = 0, red) and with increased levels of *a* (5 nS, violet and 10 nS, dark blue). Maxima and minima of oscillating *r*_ℰ_ are shown by dashed lines. Bottom: Corresponding frequencies *f*. *b* = 0.05 nA, τ_*w*_ = 200 ms, *g* = 1, and other parameter values as in Figure 2A. **(B)**: Maxima and minima of *r*_ℰ_ (top) and oscillation frequency as a function of the adaptation time constant τ_*w*_. *a* = 0, *r*^ℰ^_ext_ = 6.25 Hz, and other parameter values as in **(A)**.

Next, we investigate how these oscillations are affected by the external input *r*^ℰ^_ext_, the subthreshold adaptation conductance *a* and the adaptation timescale τ_*w*_, see Figure 3. The existence of adaptation-induced oscillations is quite sensitive to the level of *r*^ℰ^_ext_ (Figure 3A). Periodic activity is stable for small values of *r*^ℰ^_ext_ (above threshold). While oscillation frequencies increase monotonically with increasing *r*^ℰ^_ext_, oscillation amplitudes increase initially for a small interval of *r*^ℰ^_ext_ values and decrease over the following interval. For larger values of *r*^ℰ^_ext_ oscillatory activity is destabilized and asynchronous states occur. Interestingly, an increase in *a* does not lead to oscillations. On the contrary, periodic population bursts are destabilized by *a*. The dependence of oscillation amplitude and frequency on τ_*w*_ is shown in Figure 3B. Stable oscillations exist for a large range of values of τ_*w*_, where the frequencies decrease with increasing τ_*w*_. Oscillations are unstable for small adaptation timescales in the range of the membrane time constant and for very large values of τ_*w*_.

### Adaptation modulates frequencies of network-based oscillations

**Figure 4:**
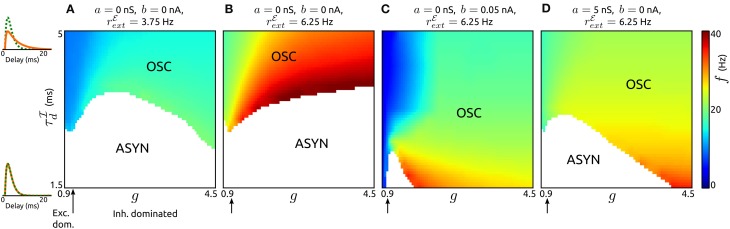
**Influence of synaptic inhibition and adaptation on network-based oscillations. (A–D)**: Existence of oscillatory states (OSC) and corresponding frequencies *f* as a function of the strength *g* and timescale τ^ℐ^_*d*_ of synaptic inhibition for networks with adaptation parameters and external input strengths as specified. Asynchronous states (ASYN) are indicated by white regions in the parameter space. Arrows mark balance of recurrent excitation and inhibition. On the left *p*^ℰ^_*d*_ (green) and *p*^ℐ^_*d*_ (orange) are shown for τ^ℐ^_*d*_ = 1.5 ms, τ^ℐ^_*d*_ = 5 ms. τ^ℐ^_*r*_ was chosen such that the peaks of *p*^ℰ^_*d*_ and *p*^ℐ^_*d*_ occur at the same delay value. τ_*w*_ = 200 ms, τ^ℰ^_*r*_ = 1.25 ms, and τ^ℰ^_*d*_ = 1.5 ms. For other parameter values see the Methods section.

Here we study the influence of adaptation on oscillations generated by recurrent synaptic excitation-inhibition (ℰ−ℐ) loops. The pace of such oscillations is believed to be largely determined by the decay of inhibition. To describe their dependence on the timescale of inhibition for various recurrent network regimes (from excitation dominated to inhibition dominated) we first consider networks of neurons without an adaptation current (*a* = *b* = 0), see Figures 4A,B. By varying the decay τ^ℐ^_*d*_ of inhibition and its strength (by parameter *g*) across networks we find that stable oscillatory states occur if inhibition is sufficiently slow in comparison to excitation. The oscillation frequencies increase with increasing external input spike rate *r*^ℰ^_ext_, increasing *g* and decreasing τ^ℐ^_*d*_, respectively. A low value of *r*^ℰ^_ext_ leads to frequencies in the low beta band (Figure 4A), for a higher value of *r*^ℰ^_ext_ the frequencies span the beta and low gamma bands (Figure 4B). Note that the network parameters can be adjusted to obtain higher oscillation frequencies. The generating mechanism underlying the oscillations is a loop of recurrent synaptic excitation and inhibition, initiated by the excitatory external input. We verified this by removing the recurrent excitatory input to the inhibitory population, which lead to a destabilization of the oscillations. For larger values of *g* as the ones used in Figure 4, the ℰ−ℐ-loop mechanism is replaced by an ℐ-ℐ-loop that does not depend on recurrent excitation (not shown). Since adaptation is only exhibited by excitatory neurons, we disregard the parameter space where ℐ-ℐ-loop-based rhythmic activity occurs and focus on ℰ-ℐ-loop-based oscillations instead.

An increase of spike-triggered adaptation or subthreshold current stabilizes oscillatory states also for faster recurrent inhibition, see Figures 4C,D. This change in single neuron dynamics causes oscillations in large parts of explored (*g*, τ^ℐ^_*d*_)-space. In particular, for spike-triggered adaptation asynchronous states only occur in a small region of the parameter space. Interestingly, the oscillation frequencies are significantly reduced by either type of adaptation.

**Figure 5:**
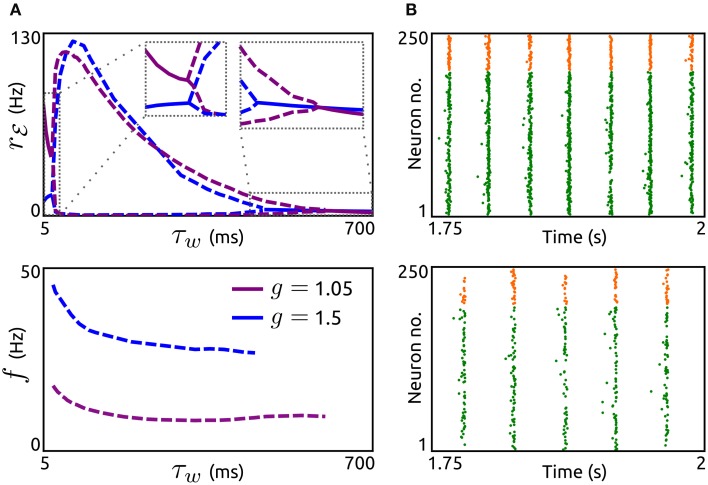
**Effects of adaptation timescale on network-based oscillations. (A)** Top: Spike rate *r*_ℰ_ as a function of the adaptation time constant τ_*w*_ for networks with dominant recurrent excitation (*g* = 1.05, violet) and inhibition (*g* = 1.5, blue). Dashed lines indicate maxima and minima of oscillating *r*_ℰ_, solid lines represent constant *r*_ℰ_. Bottom: Corresponding oscillation frequencies *f*. *a* = 5 nS and *b* = 0.05 nA. *r*^ℰ^_ext_ = 7.5 Hz, τ^ℰ^_*r*_ = 1.25 ms, τ^ℰ^_*d*_ = 1.55 ms, τ^ℐ^_*r*_ = 0.98 ms, and τ^ℐ^_*d*_ = 2 ms. Other parameters as in Figure 4. **(B)**: Raster plots of simulated networks of size *N* = 50,000 with *g* = 1.5 and τ_*w*_ = 100 ms (top) as well as τ_*w*_ = 400 ms (bottom), showing the spike times of 200 excitatory and 50 inhibitory aEIF neurons. Other parameter values as in **(A)**.

Next, we investigate how the timescale of adaptation τ_*w*_ affects oscillations mediated by an ℰ-ℐ-loop. In Figure 5A we show the dependence of amplitude and frequency of such oscillations on τ_*w*_ for networks with both adaptation components increased (*a* = 5 nS, *b* = 0.05 nA) and either dominant recurrent excitation (*g* = 1.05) or inhibition (*g* = 1.5). In both cases, stable oscillatory states exist for a large range of time constants. As τ_*w*_ increases the oscillation frequencies decrease while the amplitudes first increase abruptly and then decrease. The networks settle into asynchronous states for small τ_*w*_ (in the order of the membrane time constant) or large τ_*w*_ (several hundreds of milliseconds). Note that these effects of τ_*w*_ are similar if either *a* or *b* is increased individually (not shown). We validated these effects by simulations of aEIF neuron networks, see Figure 5B. The raster plots show that an increase in τ_*w*_ leads to a decrease in oscillation frequency and amplitude.

### Adaptation promotes periodic signal propagation

**Figure 6:**
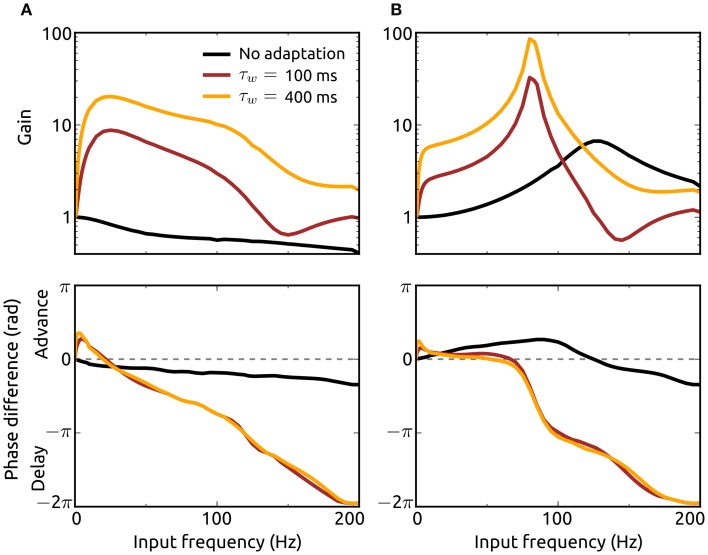
**Effects of adaptation on resonance properties of recurrent networks.** Gain (top) and phase shift (bottom) of the spike rate *r*_ℰ_ for networks with dominant recurrent excitation [*g* = 1.05, **(A)**] and inhibition [*g* = 1.5, **(B)**] as a function of the input frequency *f*. The gain is defined as the quotient of the oscillation amplitude in *r*_ℰ_ for the input with frequency *f* and the amplitude for the lowest frequency (*f*_min_ = 0.5 Hz). Adaptation parameter values are *a* = *b* = 0 (black), *a* = 5 nS, *b* = 0.05 nA, τ_*w*_ = 100 ms (dark red), and *a* = 5 nS, *b* = 0.05 nA, τ_*w*_ = 400 ms (orange). Delay distributions are identically parameterized (*p*^ℰ^_*d*_ ≡ *p*^ℐ^_*d*_) with τ_*r*_ = 1.25 ms and τ_*d*_ = 1.5 ms. The Poisson-input rates *r*^ℰ^_ext_, *r*^ℐ^_ext_ each consist of a baseline rate plus a sinusoidal component of small amplitude (1/1000th of the baseline) with frequency *f*. The baseline of *r*^ℰ^_ext_ is chosen to yield a steady-state spike rate *r*^∞^_ℰ_ of 50 Hz with constant input rate. The baseline rate of *r*^ℐ^_ext_ is chosen as explained in the Methods section.

To analyze how the resonance properties of recurrent networks in asynchronous states are influenced by adaptation currents, we here consider external Poisson-inputs with oscillatory rates with frequency *f*. Gain of input spike rate and phase difference between network and input spike rates as a function of input frequency for networks without (*a* = *b* = 0) and with adaptation (*a* = 5 nS, *b* = 0.05 nA) considering two adaptation time constants are presented in Figures 6A,B. Excitation dominated networks without adaptation do not exhibit resonance at any frequency and show only phase delays. The presence of an adaptation current leads to a significant amplification of oscillations in the input which is particularly strong at lower frequencies (of the beta band). This effect is pronounced for an increased adaptation timescale. In addition, adaptation causes a phase advance for low oscillation frequencies.

In networks where recurrent inhibition dominates excitation on the other hand even in the absence of adaptation currents resonance is shown for a high frequency band and phase advances for lower frequencies. Adaptation greatly enhances resonance and shifts the preferred frequency band to the high gamma range. The resonance effect is even stronger if the adaptation current is slower, i.e., τ_*w*_ increased. Although these effects of adaptation on resonance properties of recurrent networks are similar when either the subthreshold (*a*) or spike-triggered adaptation component (*b*) is increased individually, the dominant contribution to the frequency amplifications comes from *b* (not shown). We additionally examined the response of single neurons to oscillatory noisy inputs using our mean-field model and found that adaptation mediates resonance even in the absence of recurrent input (not shown). These results emphasize the importance of adaptation for the amplification and thus propagation of oscillatory signals in neuronal networks.

## Discussion

In this work we have investigated the role of neuronal adaptation currents in shaping spike rate oscillations in large recurrent networks of excitatory and inhibitory neurons. Based on a network of aEIF model neurons sparsely coupled through conductance-based synapses with heterogeneous delays and strengths driven by noisy external input, we used a mean-field method taking advantage of the FP equation. We simplified the problem by applying an adiabatic approximation and solved the resulting equations numerically. Using this method we obtain membrane potential distributions and population averages of spike rates and adaptation currents. At the same time, the dynamical properties of single neurons, i.e., the neuron model parameters, are retained in the derived mean-field network model.

Alternative mean-field methods have been developed for conductance-based model neurons (Robinson et al., 2008) and recurrent networks thereof in asynchronous states (Shriki et al., 2003), where spike rates are obtained without having to solve a PDE. Our approach based on the FP equation on the other hand treats noise in the synaptic inputs in more detail and allows for the calculation of membrane potential distributions in addition to spike rates.

We chose the aEIF model because it provides a rich yet low-dimensional description of neuronal dynamics and includes a proper phenomenological description of the M and AHP adaptation currents. The effects of subthreshold (*a*) and spike-triggered adaptation (*b*) on response properties of aEIF neurons (measured by spike rate-input current relationships and phase response curves) match those of M and AHP adaptation currents in a Hodgkin–Huxley type neuron model, respectively (Ladenbauer et al., 2012). Furthermore, fitting the aEIF model parameters to a detailed biophysical model using standard electro-physiological paradigms revealed a clear relationship between parameter *a* and the conductance for the M current as well as between parameter *b* and the AHP current (not shown).

Our method is based on several assumptions which allow to derive the mean-field equations. The Poisson approximation of spike train statistics is justified by experimental findings (Tolhurst et al., 1983; McAdams and Maunsell, 1999) although spiking seems to be more regular in some cortical areas (Maimon and Assad, 2009). The sparse random connectivity implies vanishing noise correlations between neurons in the large network limit and an experimental study in primary visual cortex of awake monkeys has reported almost zero noise correlations (Ecker et al., 2010). However, there is an ongoing debate about the strength of correlations in experimental data (Cohen and Kohn, 2011). We have used an adiabatic approximation, which relies on separable time scales of adaptation current and membrane voltage. Although this assumption is violated for small values of τ_*w*_, numerically solving the unreduced FP system, Equations (18)–(24), showed that our results are robust regarding the violation of this assumption. The results we obtained by simulations of aEIF networks and the mean-field results show quantitative differences. However, the presented effects described using the mean-field model are validated qualitatively by the network simulations.

We have shown that spike-triggered adaptation provides a mechanism to generate spike rate oscillations in a low frequency range (alpha band and lower) if recurrent excitation is sufficiently strong. Increased subthreshold adaptation on the other hand does not contribute to this mechanism but rather dampens such oscillations. The type of adaptation current therefore strongly determines rhythmic activity in excitation dominated networks. The importance of activity-driven adaptation for slow oscillations is consistent with results from simulations of detailed (thalamo-)cortical spiking neuron network models (Bazhenov et al., 2002; Compte et al., 2003; Destexhe, 2009), mean-field studies based on networks of excitatory neurons under the assumption sparse (Gigante et al., 2007b) and all-to-all connectivity (Nesse et al., 2008), as well as phenomenological rate models (Latham et al., 2000). We have further shown that reducing inhibitory synaptic strength leads to a reduction on oscillation frequency, which is in agreement with similar experimental findings (Sanchez-Vives et al., 2010).

The M and AHP K^+^ currents, which mediate spike frequency adaptation in pyramidal neurons, are known to be deactivated by acetylcholine (McCormick, 1992), with the AHP current showing higher sensitivity. Since the adaptation parameter *b* is strongly related to AHP type adaptation, our results support the hypothesis that the cholinergically induced activating transition from slow-wave oscillations to asynchronous irregular states (Lee and Dan, 2012) is mediated (at least in part) by a reduction of spike-triggered adaptation (Destexhe, 2009).

We have demonstrated that an increase of either type of adaptation current leads to a reduction in the frequency of oscillations generated by a loop of recurrent excitation and inhibition. This shows that the dynamical properties of neurons in addition to coupling characteristics strongly affect the network frequency. Also the passive (integrative) membrane properties significantly influence such networks oscillations as has been described previously (Geisler et al., 2005). Our additional finding of decreased frequencies for increased adaptation time constants is consistent with the results from a computational study on clustering effects of spike-triggered adaptation in gamma oscillations (Kilpatrick and Ermentrout, 2011).

Low input frequencies have been shown to be suppressed in the output of single excitatory neurons with increased spike-triggered (Gigante et al., 2007a) or subthreshold adaptation (Richardson et al., 2003; Prescott and Sejnowski, 2008), which we confirmed using our aEIF-based mean-field model. Such a high pass property of single neurons has also been found using a more general model of adaptation (Benda and Herz, 2003). We have demonstrated that both adaptation currents cause spike rate resonance in excitation dominated recurrent networks. Inhibition dominated networks, on the other hand, exhibit resonance without adaptation and we have shown that increased adaptation of excitatory neurons strongly amplifies this resonance. A similar effect has been described for purely inhibitory networks (Richardson, 2009). In addition, our results show that adaptation shifts the resonance frequency to lower values.

In excitation dominated networks, adaptation further leads to phase advances for low input frequencies in addition to phase delays for higher frequencies as observed in previous studies on single excitatory neurons (Fuhrmann et al., 2002; Gigante et al., 2007a). These adaptation-induced phase advances enable synchronization of periodic activity between distant neurons (and populations of neurons) in different areas of the brain if the strength of adaptation is controlled appropriately, e.g., through cholinergic neuromodulation.

Here we have considered one adaptation current for each neuron of the excitatory population. To account for the multimodal distribution of adaptation timescales found experimentally (La Camera et al., 2006) our approach can be easily extended to include multiple adaptation currents.

### Conflict of interest statement

The authors declare that the research was conducted in the absence of any commercial or financial relationships that could be construed as a potential conflict of interest.
